# SOST/Sclerostin impairs the osteogenesis and angiogesis in glucocorticoid-associated osteonecrosis of femoral head

**DOI:** 10.1186/s10020-024-00933-5

**Published:** 2024-09-28

**Authors:** Junming Huang, Tianle Ma, Chenzhong Wang, Zhe Wang, Xinyuan Wang, Bingxuan Hua, Chang Jiang, Zuoqin Yan

**Affiliations:** Department of Orthopaedics, Zhongshan Hospital, Fudan University, 180 Feng Lin Road, Xuhui District, Shanghai, 200032 China

**Keywords:** Osteonecrosis of femoral head (ONFH), Osteocytes, Sclerostin (SOST), Osteogenesis, Angiogenesis

## Abstract

**Background:**

Glucocorticoid-associated osteonecrosis of the femoral head (GA-ONFH) is a progressive bone disorder which frequently results in femoral head collapse and hip joint dysfunction. Sclerostin (SOST) is principally secreted by osteocytes in bone and plays an important role in bone homeostasis and homeostasis of skeletal integrity. Our previous study reported that short‐term use of glucocorticoid increased serum sclerostin levels. Here this study is aimed to identify whether sclerostin played an essential role in the occurrence and development of GA-ONFH.

**Methods:**

Glucocorticoid-induced osteonecrosis of femoral head (ARCO stage II) samples were collected and sclerostin staining was conducted. Osteocyte cell line Ocy454, MC3T3-E1 and endothelial cells was used. MC3T3-E1 or endothelial cells were co-cultured with Ocy454 or SOST-silencing Ocy454 in presence of dexamethasone to mimic the crosstalk of various cells in the bone niche. GA-ONFH rat model and SOST knockout model was built to better understand the phenomenon in vivo.

**Results:**

Sclerostin was highly concentrated in osteonecrosis patient sample in the necrotic area. Co-culture with osteocytes aggravated the inhibition of dexamethasone on MC3T3-E1 and endothelial cells. Sclerostin derived from osteocytes impaired osteogenesis and angiogenesis via inhibiting the Wnt pathway. In GA-ONFH rat model, SOST knockout ameliorated the incidence of osteonecrosis and improved bone metabolism compared with the wild type group through histological, immunohistochemical and bone metabolic analyses.

**Conclusion:**

Sclerostin contribute to pathologic process of GA-ONFH by impairing osteogenesis and angiogenesis.

**Supplementary Information:**

The online version contains supplementary material available at 10.1186/s10020-024-00933-5.

## Background

Glucocorticoids (GC) are economically-efficient immunomodulators and has been widely used clinically to treat various acute and chronic condition, especially rheumatic, auto-immune and hematopoietic diseases. In clinical practice however, high-dose and/or long-term use of GC may result in a variety of complications, one of the more serious complications being osteonecrosis of the femoral head (ONFH). The specific global prevalence of glucocorticoid-associated ONFH (GA-ONFH) has not been reported, but is assumed that 10,000–20000 new cases of ONFH are identified annually in the United States, and the most common risk factor is the usage of GC (51%) (Gagala et al. 2013; Wang et al. 2018). Young and male patients between 30 and 50 years old, are more susceptible to GA-ONFH (Lieberman et al. 2003; Arbab and König 2016; Cui et al. 2016). As a devastating, irreversible and disabling orthopedic disease, GA-ONFH significantly affects the quality of life and is a huge economic burden to society. However, effective therapeutic methods in clinical practice are limited. Therefore, it is necessary to identify the pathogenesis of GA-ONFH and then explore potential therapeutic modalities.

Traditionally, ischemia is regarded to be the main etiological factor in the development of GA-ONFH. Usage of GC leads to endothelial cells dysfunction and damage, resulting in vasoconstriction, thrombus formation in the femoral head impairing blood flow to the femoral head and compromise microcirculation (Kerachian et al. 2010). GC administration is also strongly associated with coagulation abnormalities, hyperlipidemia and platelet activation, resulting in a hypercoagulable state which is followed by impaired blood flow, ischemia, and eventually ONFH (Zhang 2018). GC-induced bone metabolic changes are also considered as a contributing factor in the development of GA-ONFH. A previous study reported that downregulated telomerase activity and the self-renewal ability induced by GC usage inhibit cellular proliferation and osteogenic differentiation of bone marrow stem cells (BMSCs) (Zhang, et al. 2016). GCs are also known to inhibit osteoblastic proliferation and differentiation, and aggravate osteoblasts apoptosis.

Osteocytes are the major bone cells essential for maintaining bone homeostasis via orchestrating growth, maintenance, and healing. In bone niche, osteocytes play an active role in regulating bone metabolism through the regulation of paracrine and endocrine secretions including sclerostin (Compton 2014). Previous studies demonstrated that Sclerostin (SOST) plays an important role in the maintenance of bone homeostasis and skeletal integrity. Dysregulation of sclerostin commonly manifests as an abnormally increased bone mass characterized by exaggerated bone formation, including sclerosteosis, van Buchem disease, and craniodiaphyseal dysplasia (Balemans, et al. 2002; Balemans, et al. 2001; Staehling-Hampton, et al. 2002; Brunkow, et al. 2001). In osteoporotic patients, serum level of sclerostin was increased and associated with increased fracture risk. A previous clinical trial have reported that administration of antibodies to sclerostin (Scl-Ab) resulted in increased bone mass and decreased fracture risks (Huybrechts et al. 2020). To our knowledge however, there is no study reporting the role of sclerostin in GA-ONFH. Our previous work showed that serum concentrations of sclerostin was increased after short‐term use of GCs (Wang et al. 2019). Considering sclerostin as a negative regulator in bone homeostasis, we speculate that the increased sclerostin contribute to the development of GA-ONFH. The purpose of this study was to assess whether SOST defects can prevent the development of GA-ONFH in rat model and to explore its potential molecular mechanism.

## Materials and methods

### Specimens collection

Glucocorticoid-induced osteonecrosis of femoral head (ARCO stage II) samples were collected from a patient who received bone grafting in Zhongshan Hospital, Fudan University (Shanghai, China). The patient with ONFH had a medical history of the steroid administration owing to systemic lupus erythematosus (SLE). This study was approved by the Ethical Committee of the Zhongshan Hospital (B2019-135R) and informed content was obtained from the patient.

### Reagents

Fetal bovine serum (FBS), penicillin and streptomycin were provided by Gibco Life Technologies (Grand Island, NY, USA). α-Modified Eagle’s Medium (α-MEM) was purchased from Hyclone (Waltham, MA, USA). Endothelial Cell Medium (ECM) was purchased from ScienCell (Carlsbad, CA, USA). Osteogenic differentiation medium of MC3T3-E1 Cell was purchased from Cyagen (Santa Clara, CA, USA). Matrigel was purchased from Corning (New York, USA). Phalloidin was provided by Beyotime (Shanghai, China). Cy3- conjugated goat anti-rabbit secondary antibody (BA1032) and 4’, 6-Diamidino-2-phenylindole (DAPI) (AR1177) were purchased from Boster (Wuhan, China). Mouse SOST Elisa Kit (ELK 6089) was purchased from ELK Biotech (Wuhan, China). Dexamethasone (CAS No. 50-02-2) and other reagents were of the highest commercial grade and were purchased from Sigma Chemical (St. Louis, MO, USA).

### Cell culture

Murine osteocyte (Ocy454) was provided by Prof. Pajevic (Massachusetts General Hospital and Harvard Medical School). Murine Osteoblast cell line (MC3T3-E1) was purchased from ATCC. Murine arterial vascular endothelial cells were isolated from previous study (Wang et al. 2016). Ocy454 and MC3T3-E1 were cultured in α-MEM contained 10% FBS, 100 U/mL penicillin and 100 mg/mL streptomycin. Vascular endothelial cells were cultured in ECM contained 5% FBS, 5 ml endothelial cell growth supplement, 100 U/mL penicillin and 100 mg/mL streptomycin. Ocy454, MC3T3-E1 and vascular endothelial cells were maintained in humid incubator with 5% CO2 at 37 °C. The medium was replaced every 2 days and passaged upon 80%–90% confluency. To investigate the role of sclerostin in GA-ONFH, Trans-well chamber system was used for co-culture experiment. MC3T3-E1 or vascular endothelial cells were cultured in the lower chamber with corresponding medium. Ocy454 or SOST-silencing Ocy454 were seated in the upper wells with α-MEM containing dexamethasone.

### Cell viability assay

A cell counting kit (CCK, Dojindo, Japan) was used to analyze cell viability in the co-culture experiment. Different groups of MC3T3-E1 or vascular endothelial cells were seeded at density 1 × 10^4^ cells/well. To measure cell viability, 10 μL CCK-8 solution and 90 μL medium were added to each well, and then, the plates were incubated in the dark at 37 °C for 1.5 h. The absorbance values of the supernatants were recorded in 450 nm.

### ELISA assay

In co-culture system, MC3T3-E1 or vascular endothelial cells were passaged at 80% confluence, cells were washed, and fresh medium was added. After 24 h, the supernatant was collected, and Sclerostin levels were measured using mouse SOST Elisa kit as per the instruction manual.

### Alizarin red (ALR) staining

MC3T3-E1 were seeded onto 24-well plates at a density of 1 × 10^5^ cells/well and incubated for 21 days in an osteogenic differentiation medium. Upon completion of osteogenic differentiation, cells were washed with PBS twice and fixed in 4% paraformaldehyde for 30 min at room temperature. After fixation, cells were treated with 1 mL alizarin red staining solution for 3–5 min and gently rinsed by tri-distilled water before analyzed under a light microscope.

### Tube formation assay

In tube formation assay, 200ul of Matrigel was coated in the lower chamber and the vascular endothelial cells were seeded on the Matrigel at density of 5 × 10^4^ cells/well. After the plate was placed into an incubator for 1 h, tube formation was monitored using a microscope 6 h after inoculation.

### Total RNA extraction and quantitative real-time RT-PCR

Total RNA was extracted by total RNA extraction kit in accordance with the manufacturer’s instructions, OMEGA. The purity and concentration of the RNA were determined by a spectrophotometer (Thermo Fisher Scientific, USA). Complementary DNA (cDNA) was synthesized from total RNA and amplified with SYBR Green Master Mix in an ABI PRISM 7500 PCR Sequence Detection System (Applied Biosystems, Foster City, CA, USA) according to following condition: 30 s of denaturation followed by 40 cycles of 94 °C for 5 s and 60 °C for 35 s. The melting curve was generated to test for primer dimer formation and false priming for each reaction. Relative expression of gene-specific products was analyzed using the comparative Ct (2 − ΔΔCt) method and normalized to the reference gene β-actin.

### Western blotting analysis

The total proteins were obtained from different groups of MC3T3-E1 or vascular endothelial cells by RIPA lysis buffer containing 1% proteinase inhibitor and 1% phosphatase inhibitors cocktail for 30 min on ice at the indicated time points. The concentration of protein was measured using the BCA protein assay kit (Boster, Wuhan, China). Then, 20 μg of protein was separated resolved on 10% SDS-PAGE gels and transferred to PVDF membrane (Millipore), blocked with 5% BSA in TBS-T (0.1% Tween-20) and incubated with primary antibody (2% BSA in TBS-T) overnight at 4 °C. Subsequently, the membrane was washed with TBS-T and incubated the corresponding secondary antibodies for 2 h at room temperature. Finally, the protein bands were visualized through Western ECL Substrate Kit (Yseasen, Shanghai, China) on Tanon imaging system and grayscale was analyzed with ImageJ software. Antibodies against phospho-GSK-3β (8213), GSK-3β (12,456), β-catenin (9562), non-phospho (Active)-β-catenin (8814), Connexin 43 (3512) were bought from Cell Signaling Technology (Beverly, MA, USA). Antibodies against ALP (ab229126), Runx2 (ab192256), Sclerostin (ab63097), VEGF (ab1316) were bought from Abcam (Cambridge, UK). Antibodies against VEGF and β-actin were bought from Proteintech Group (Wuhan, China).

### Small interfering RNA (siRNA) assays

In line with the manufacturer’s protocol, osteocyte (Ocy454) was transfected with 20 μM siRNAs by using siRNA Transfection Reagent (Ribbio, Guangzhou, China). After 2 days of cultivation, transfected osteocytes were harvested for subsequent experiment. siRNAs used for siRNA assays: si-1 CTGAGAACAACCAGACCAT; si-2 ATCCCTATGACGCCAAAGA; si-3 ACACCCGCTTCCTGACAGA.

### Animal model

In our study, all experimental procedures on the animals were in accord with the Ethics Committee on Animal Experimentation of Zhongshan Hospital, Fudan University (Shanghai, China). All rats were kept in the animal care facility of Zhongshan Hospital. The living environment of mice were maintained at 25 °C with 12:12 light/dark cycle, and mice were fed with normal chow and water. SOST-Knockout (SOST ^−/−^) rats were generated using the CRISPR/Cas9 system as previously decribed (Bäck et al. 2019; Ma et al. 2014). We designed two single guide RNA (sgRNA) targeting exons 2 in SOST gene (NM_030584.1). The mixture of transcribed Cas9 and sgRNA was injected into Sprague–Dawley rat monocytic embryos. We obtained SOST heterozygous rats (F0) and the heterozygous rats were hybridized wide-type rat to obtain next generation of SOST heterozygous rats (F1). After genomic DNA sequencing, the F1 were inbred to produce F2 generation rats, and then the homozygous were identified by the same methods. As shown in Fig. 6c, 3-month-old wide-type rat (n = 12) and SOST-KO rats (n = 12) were injected 20 μg/kg lipopolysaccharide (LPS) intravenously for two consecutive days. One day after last injection of LPS, methylprednisolone (MPS 60 mg/kg) was injected intramuscularly in rats 3 times a week for 4 weeks. After 4 weeks, all rats were sacrificed with 4 ml 10% chloral hydrate. Before injection of LPS, the blood samples were collected and then we collected blood samples every 2 weeks. All collected samples were tested for β-CTX, CTX-I, CTX-II, OCN and 25(OH)D3 using ELISA kits by following the user manual.

### Micro-computed tomography (μ-CT) scanning and analysis

The bone tissue was scanned by Sanco viva CT40 instrument (Scanco, Brüttisellen, Switzerland) under the scanning conditions of 100 kV and 98μA. The thickness of the tomographic image was 10.5 μm. The results of bone structure measurement were analyzed according to previous literature (Huang et al. 2017a; Huang et al. 2018).

### Histological and immunohistochemical staining

All samples were collected and decalcificated in 10% tetrasodium-EDTA aqueous solution at 4 °C for 1 months. Decalcified tissue was embedded by paraffin and sectioned for hematoxylin and eosin (H&E staining) and immunohistochemical staining. In immunohistochemical staining, sections were deparaffinized, antigen retrieved, blocked and incubated with primary antibodies of ALP and VEGF and corresponding biotinylated secondary antibodies. Then sections were stained with DAB and counterstained with haematoxylin.

### Statistical analysis

The experiments were at least performed three times. All data were presented as mean ± standard deviation (SD). Statistical analyses were performed using Graph Pad Prism software and SPSS 18.0 (IBM, Armonk, USA). For differences among treatments, Student’s t-test was used for the comparisons between two groups and data involving more than two groups were analyzed by one-way ANOVA followed by Tukey post hoc test. *P* values less than 0.05 were considered statistically significant.

## Result

### Sclerostin is significantly upregulated in necrotic area of the femoral head

**Fig. 1:**
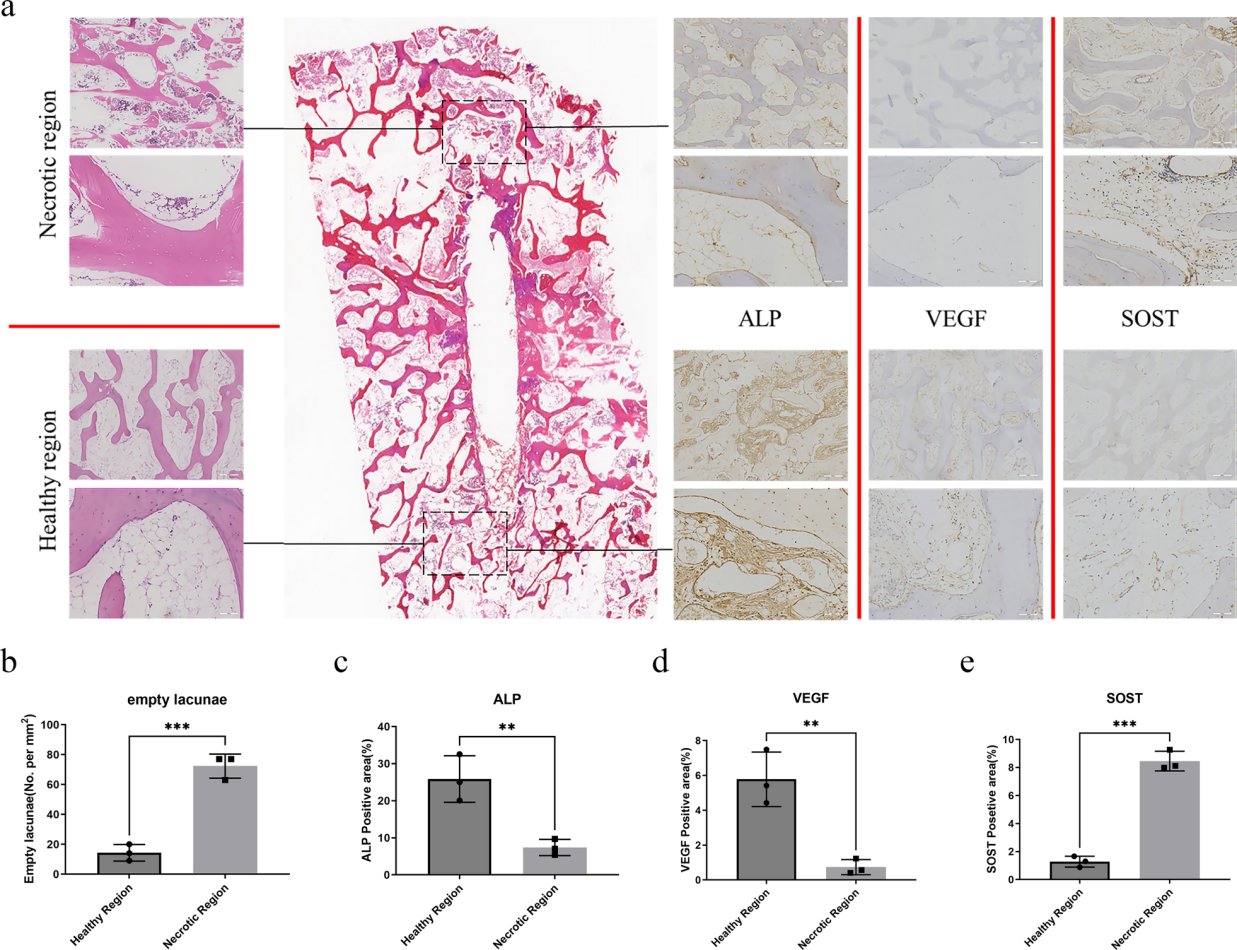
Sclerostin is significantly upregulated in necrotic area of the femoral head. **a** The gross appearance of bone sample and HE, ALP, VEGF and SOST staining of femoral head. **b**, **c**, **d**, **e** Analysis of empty osteocyte lacunae, ALP, VEGF and SOST staining showing the necrotic region is featured by the presence of empty osteocyte lacunae, weaker ALP and VEGF staining and stronger SOST staining. All data were presents as mean ± SD, n = 3, ∗∗ *P* < 0.01; ∗∗∗ *P* < 0.001

The collected osteonecrotic bone tissue was analyzed by histopathological evaluation. In necrotic region, we found diffuse empty lacunae and pyknotic nuclei in trabeculae compared with the healthy region (Fig. 1a, b). The immunohistochemical staining of ALP and VEGF was used to assess the osteogenic and angiogenic activity in bone tissue. The stanning of ALP and VEGF was significantly weaker in necrotic region compared with normal region (Fig. 1a, c, d). In addition, we examined the sclerostin expression and found the expression of sclerostin was more obvious in necrotic region (Fig. 1a, e).

### Co-culture with osteocytes aggravated the inhibition of dexamethasone on cells

**Fig. 2:**
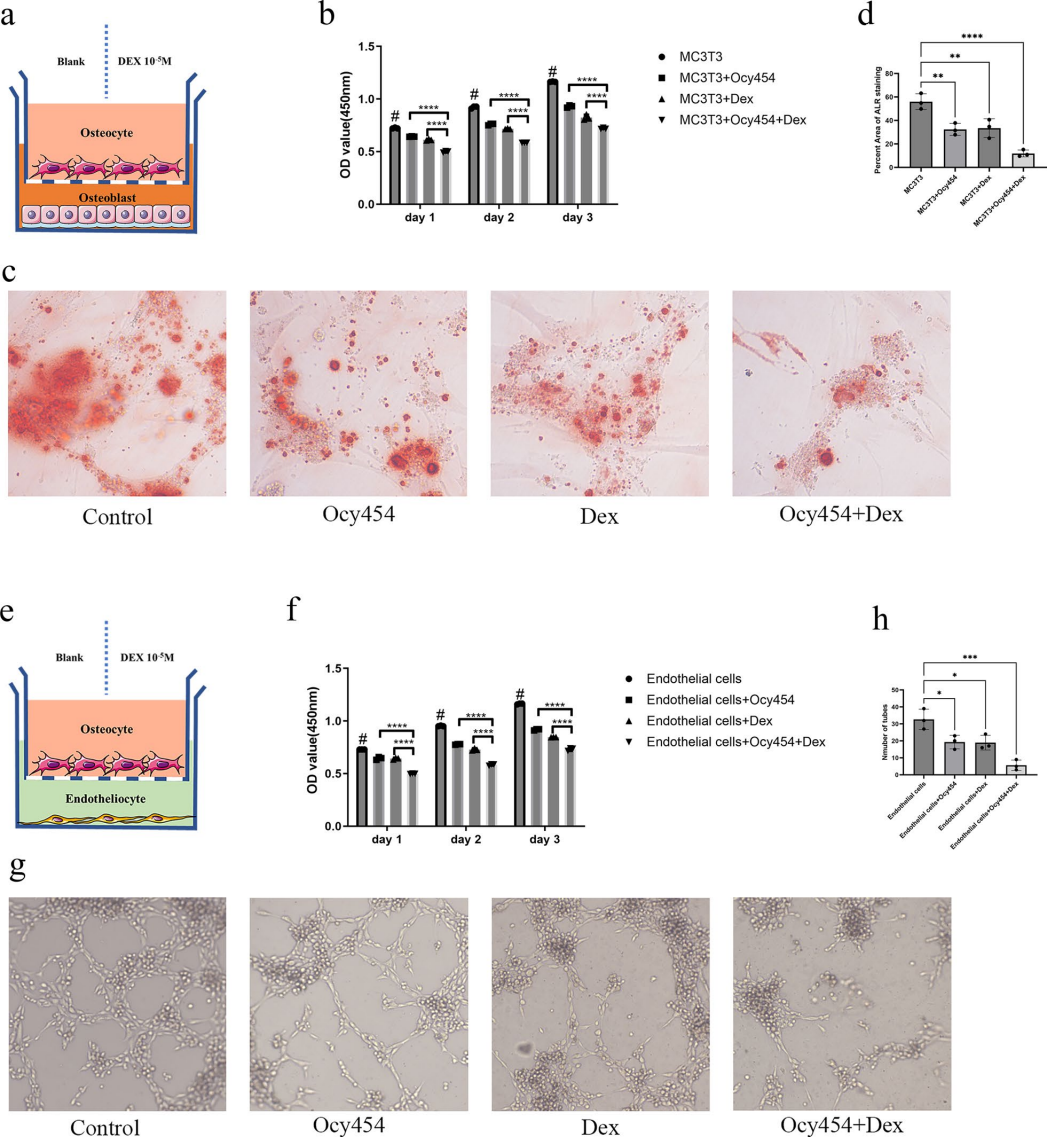
Co-culture with osteocytes aggravated the inhibition of dexamethasone on cells. **a** Schematic illustration of the co-culture system between MC3T3 and osteocytes. **b** Co-culture with osteocytes aggravated the inhibitory effect of dexamethasone on proliferation of MC3T3-E1 by CCK-8 assay. **c** Co-culture with osteocytes aggravated the inhibitory effect of dexamethasone on osteogenesis by Alizarin red staining. **d** The quantification of Alizarin red staining. **e** Schematic illustration of the co-culture system between vascular endothelial cells and osteocytes. **f** Co-culture with osteocytes aggravated the inhibitory effect of dexamethasone on proliferation of vascular endothelial cells. **g** Co-culture with osteocytes aggravated the inhibitory effect of dexamethasone on tube formation. **h** The quantification of tube formation. All data were presents as mean ± SD, n = 3, #*P* < 0.05 versus other three groups; ∗ *P* < 0.05; ∗∗ *P* < 0.01; ∗∗∗ *P* < 0.001; ****P < 0.0001

**Fig. 3:**
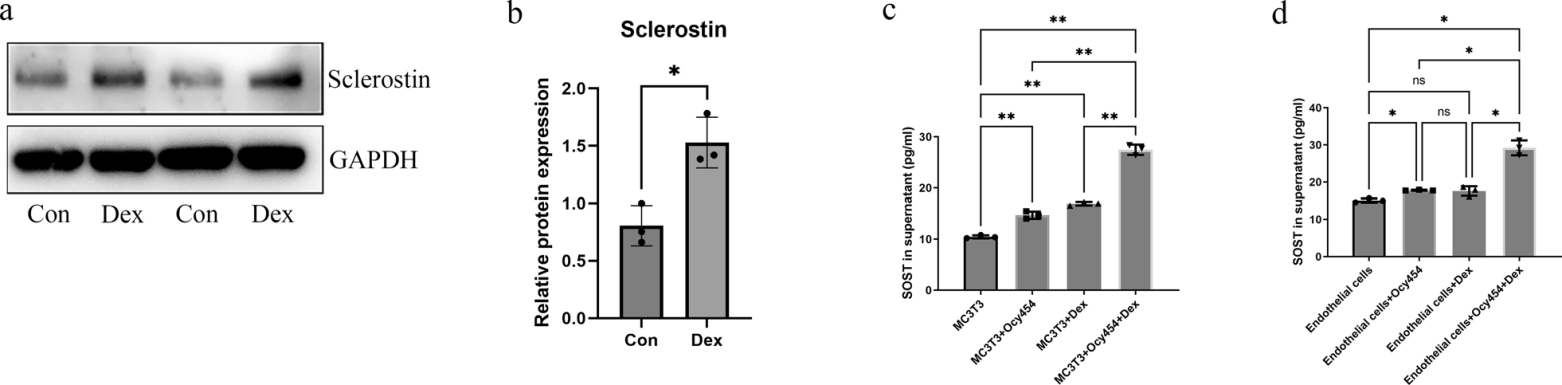
Dexamethasone stimulated sclerostin expression in osteocytes. **a**, **b** Western blot analysis showed protein expression of sclerostin in osteocytes. **c**, **d** The content of sclerostin increased after dexamethasone treatment in co-culture system. All data were presents as mean ± SD, n = 3, ns, not statistical significance; ∗ *P* < 0.05; ∗∗ *P* < 0.01

To investigate the effect of sclerostin on MC3T3-E1 or vascular endothelial cells in presence of dexamethasone, we used co-culture model of Ocy454 with MC3T3-E1 in concentration of 10^–5^ M (Fig. 2a). In CCK-8 assay, we found that co-culture with osteocytes and co-culture of osteocytes under dexamethasone treatment could inhibit the proliferation of MC3T3-E1 at day 1, 2 and 3, and the inhibitory effect of dexamethasone-treated co-culture group was the most significant (Fig. 2b). In osteogenic differentiation of MC3T3-E1, we found dexamethasone, co-culture and dexamethasone-treated co-culture could inhibit osteogenic differentiation, and the number of calcium nodules was the least in co-culture of osteocytes under dexamethasone treatment (Fig. 2c, d). For vascular endothelial cells, we observed similar phenomenon, that is, dexamethasone, co-culture and dexamethasone-treated co-culture inhibited the proliferation and vascularization of vascular endothelial cells and the most obvious inhibitory effect occurred in dexamethasone-treated co-culture group (Fig. 2e–h). In vitro, we verified the effect of dexamethasone on secretion of sclerostin from osteocytes, and found that dexamethasone stimulates the expression of sclerostin in osteocytes (Fig. 3a, b). Our elisa assay results also show that, the content of sclerostin in the supernatant of MC3T3-E1 is slightly increased in dexamethasone and co-culture groups and the content of sclerostin in dexamethasone-treated co-culture group was significantly higher than the other groups (Fig. 3c). For vascular endothelial cells, dexamethasone treatment showed no increased sclerostin in supernatant. Co-culture with osteocytes and co-culture of osteocytes under dexamethasone treatment both stimulated sclerostin expression in supernatant of vascular endothelial cells and the content of sclerostin in dexamethasone-treated co-culture group was also significantly higher than that in co-culture group (Fig. 3d). Thus, in both of MC3T3-E1 and vascular endothelial cells, the significant increase of sclerostin mainly from the stimulation of osteocytes by dexamethasone may contribute to the final outcome of dexamethasone on both cells.

### Silencing SOST of osteocytes rescued the inhibition of dexamethasone on cells in co-culture system

**Fig. 4:**
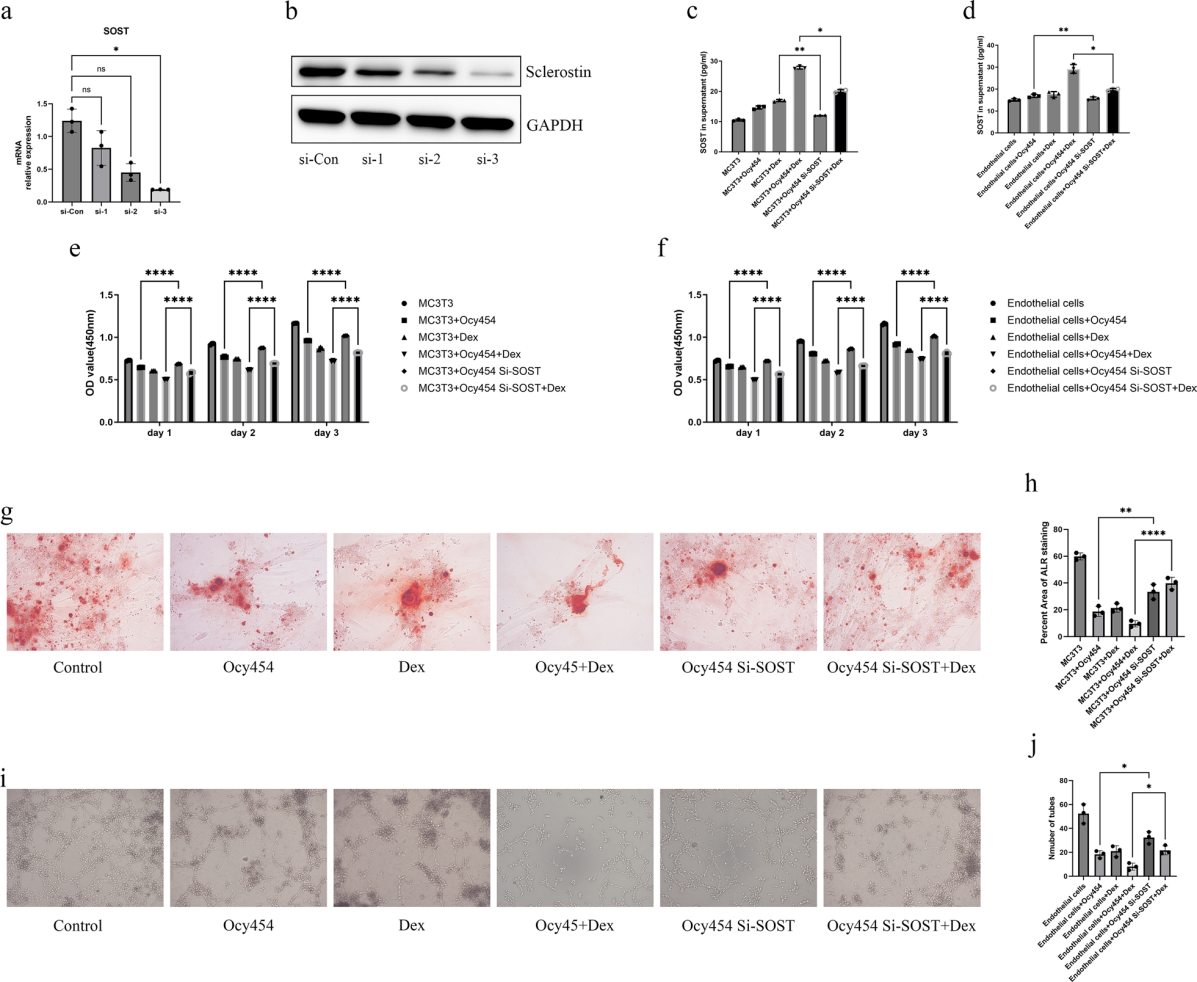
Silencing SOST of osteocytes rescued the inhibition of dexamethasone on cells in co-culture system. **a** The mRNA of SOST downregulated after siRNA transfection. **b** The protein expression level of sclerostin was downregulated after siRNA transfection. **c**, **d** The content of sclerostin was decreased after siRNA transfection in the co-culture system. **e**, **f** Silencing SOST of osteocytes antagonized the inhibitory effect of dexamethasone on proliferation of MC3T3-E1 and vascular endothelial cells. **g** Silencing SOST of osteocytes antagonized the inhibitory effect of dexamethasone on osteogenesis. **h** The quantification of Alizarin red staining. **i** Silencing SOST of osteocytes antagonized the inhibitory effect of dexamethasone on tube formation. **j** The quantification of tube formation. All data were presents as mean ± SD, n = 3, ns, not statistical significance; ∗ *P* < 0.05; ∗∗ *P* < 0.01; ****P < 0.0001

To investigate the effect of sclerostin on MC3T3-E1 or vascular endothelial cells in presence of dexamethasone, we used siRNA to silence the SOST expression in the osteocytes of the co-cultured model. The siRNA that silenced three different fragments of SOST were transfected into osteocytes and the third SOST siRNA showed the most significant silent effect which will be used as a follow-up experiment (Fig. 4a, b). In elisa assay, the content of sclerostin in the supernatant of MC3T3-E1 or vascular endothelial cells was decreased in co-culture and dexamethasone-treated co-culture groups respectively by silencing SOST of osteocytes (Fig. 4c, d). In CCK-8 assay, silencing SOST of osteocytes could rescue the proliferation of MC3T3-E1 or vascular endothelial cells in co-culture and dexamethasone-treated co-culture groups respectively at day 1, 2 and 3 (Fig. 4e, f). In osteogenic differentiation of MC3T3-E1, when SOST silenced osteocytes co-cultured with MC3T3-E1, the osteogenic differentiation was significantly better than that of normal co-culture group and we observed similar better osteogenic differentiation in group of dexamethasone-treated co-culture with SOST silenced osteocytes (Fig. 4g, h). In angiogenesis assay, silencing SOST of osteocytes rescued inhibition of tube formation in group of co-culture and dexamethasone-treated co-culture (Fig. 4i, j).

### Silencing SOST of osteocytes rescued expression osteogenic and angiogenic gene through facilitating β-catenin activation

**Fig. 5:**
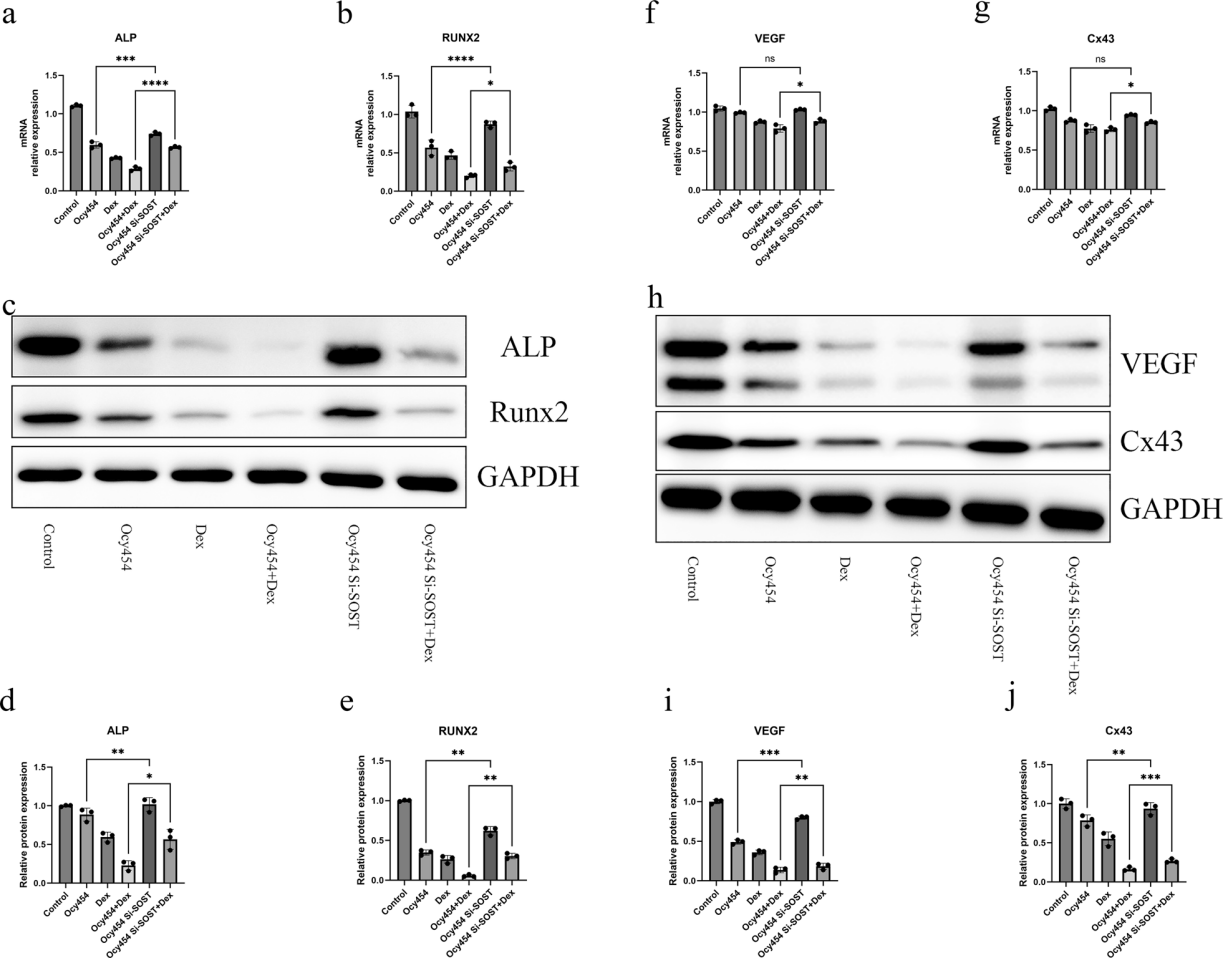
Silencing SOST of osteocytes rescued the expression of osteogenic and angiogenic genes. **a**, **b** Silencing SOST of osteocytes attenuated downregulated mRNA expression of osteogenic gene, RUNX2 and ALP, caused by dexamethasone. **c**, **d**, **e** Western blot analysis showed protein expression of Runx2 and ALP after silencing SOST of osteocytes in co-culture system. **f**, **g** Silencing SOST of osteocytes attenuated downregulated mRNA expression of angiogenic gene, Cx43 and VEGF, caused by dexamethasone. **h**, **i**, **j** Western blot analysis showed protein expression of Cx43 and VEGF after silencing SOST of osteocytes in co-culture system. All data were presents as mean ± SD, n = 3, ns, not statistical significance; ∗ *P* < 0.05; ∗∗ *P* < 0.01; ∗∗∗ *P* < 0.001; ****P < 0.0001

**Fig. 6:**
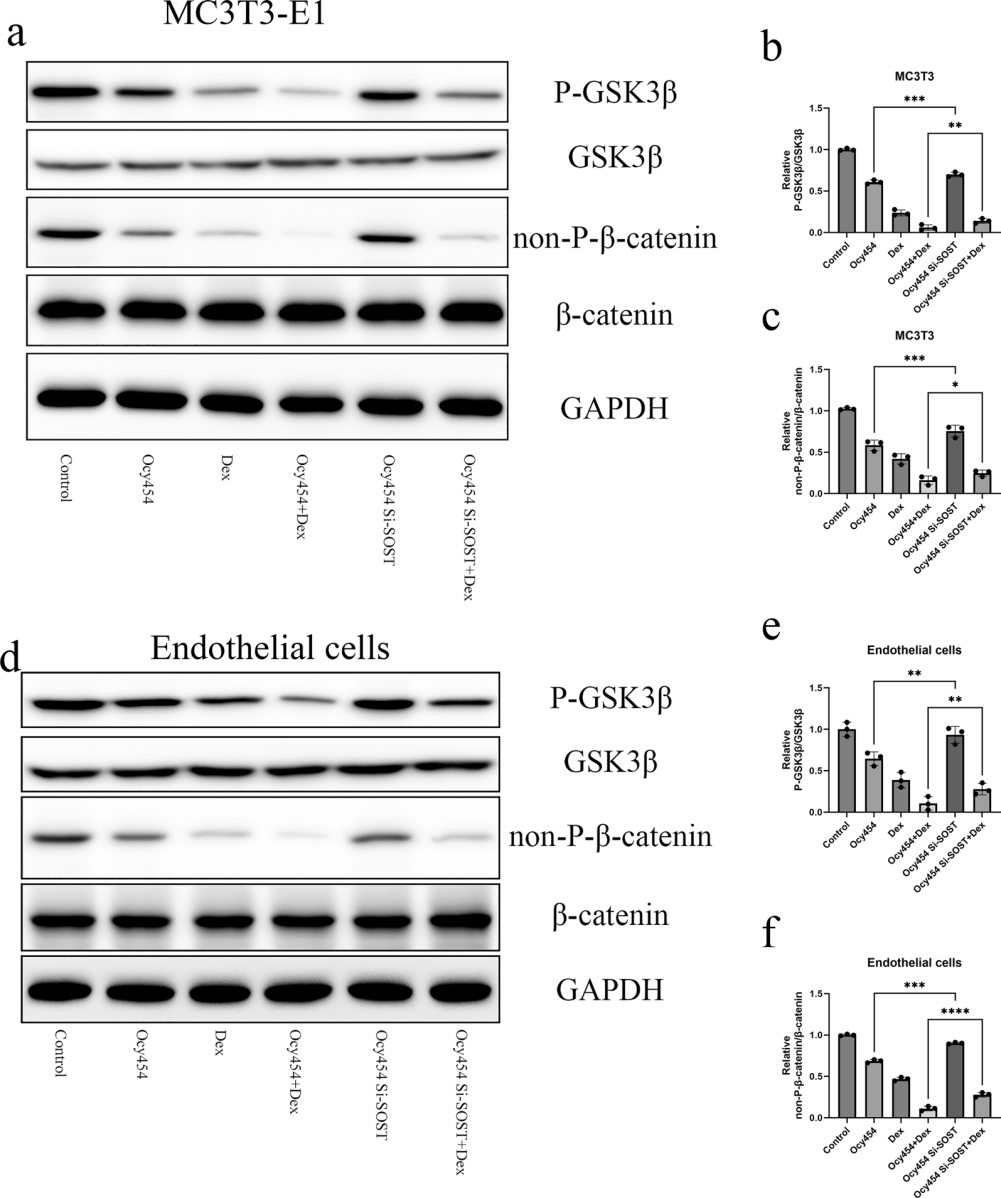
Silencing SOST of osteocytes facilitated β-catenin activation. **a**, **b**, **c** Western bolt was performed to evaluate the protein amount of P-GSK3β, GSK3β, non-P-β-actin and β-actin in MC3T3-E1. **d**, **e**, **f** Western bolt results evaluating the protein amount of P-GSK3β, GSK3β, non-P-β-actin and β-actin in vascular endothelial cells. All data were presents as mean ± SD, n = 3, ∗∗ *P* < 0.01; ∗∗∗ *P* < 0.001; ****P < 0.0001

After 7 days of osteogenic and angiogenetic induction, we collected RNA and protein from each sample for further experiment. Osteogenic-specific genes, including RUNX2 and ALP, were robustly down-regulated in dexamethasone-treated co-culture group. When the SOST of osteocytes is silenced, the decreased expression of RUNX2 and ALP was alleviated (Fig. 5a, b). Correspondingly, the protein expression of Runx2 and ALP was down-regulated most significantly in dexamethasone-treated co-culture group and the protein expression of Runx2 and ALP was partly restored through silencing SOST (Fig. 5c–e). For angiogenetic-associated genes, the RT-PCR results showed that silencing SOST barely affects the expression of VEGF and Cx43 in co-culture group, but upregulates the low expression of VEGF and Cx43 in dexamethasone-treated co-culture group (Fig. 5f, h). The western blot analyses of VEGF and Cx43 basically corroborated the mRNA findings with differences, that is, silencing SOST could also restore the expression of VEGF and Cx43 in co-culture group (Fig. 5i–k). Given that SOST is a vital inhibitor of Wnt signaling pathway, western blotting was performed to verify the protein expression in the Wnt signalling pathway during osteogenesis and angiogenesis. In MC3T3-E1, the expression of sclerostin from Ocy454 impaired the phosphorylation of GSK-3β and decreased the expression of Non-phospho (Active) β-catenin and silencing SOST could upregulate the amount of phosphorylated GSK-3β and active β-catenin (Fig. 6a–c). A similar trend was also observed in vascular endothelial cells and silencing SOST attenuated the downregulated phosphorylated GSK-3β and active β-catenin (Fig. 6d–f).

### GA-ONFH was alleviated by knockout of SOST in the rat model

**Fig. 7:**
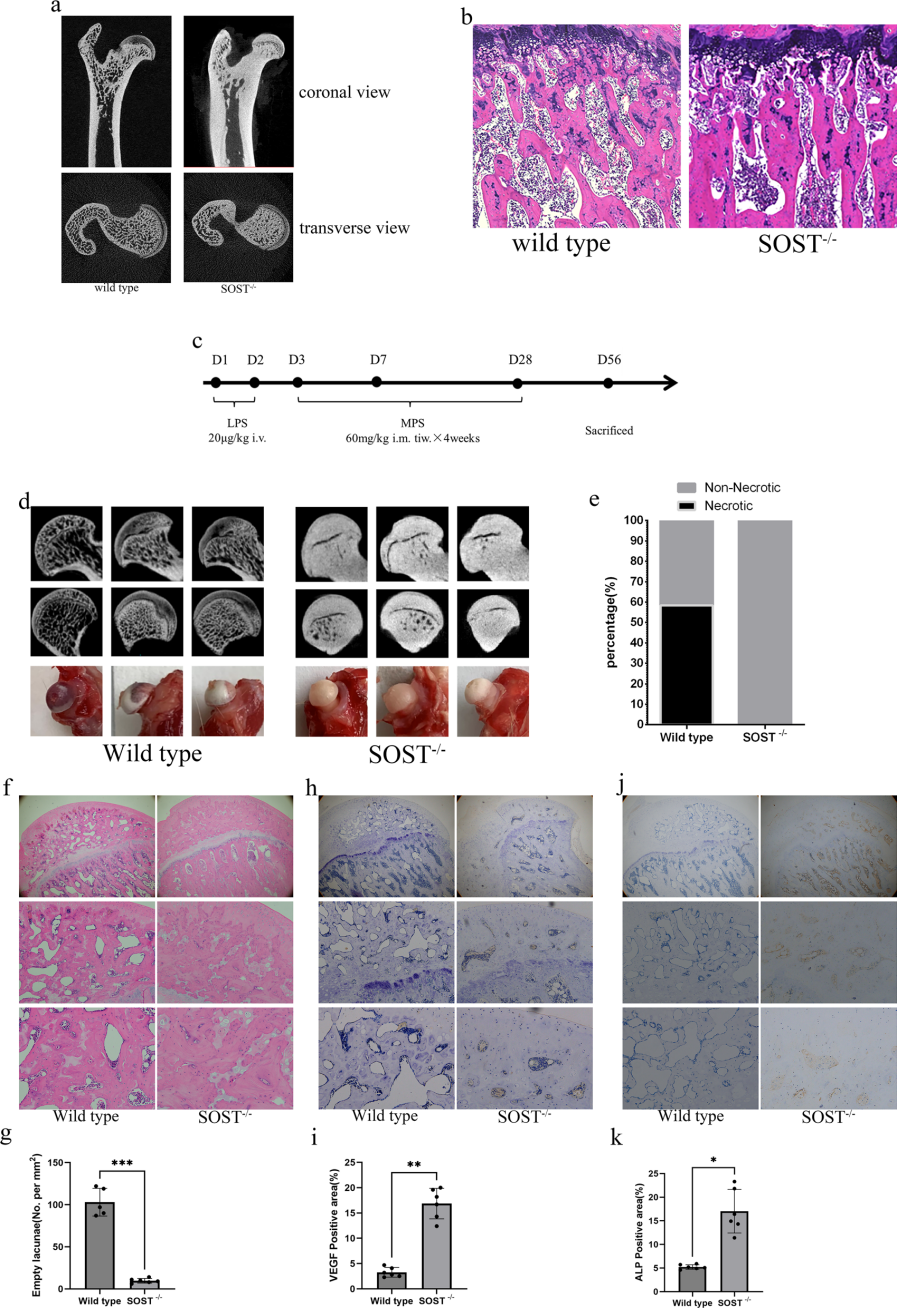
GA-ONFH was alleviated by knockout of SOST in the rat model. **a**, **b** The image of micro-CT and HE stanning of femoral head from wild type and SOST^−/−^ rats. **c** Schematic illustration of the establishment of rat ONFH model. **d** The image of micro-CT and gross appearance of femoral head from wild type and SOST^−/−^ rats after steroid administration **d** the image of micro-CT and HE stanning of femoral head from ONFH model. **e** The incidence rate of ONFH in wild type and SOST^−/−^ rats. **f**, **g** HE stanning showed that SOST knockout decrease quantity of empty lacunae **h**, **i** Immunohistochemical staining of ALP showed SOST knockout attenuate downregulation of ALP. **j**, **k** Immunohistochemical staining of VEGF showed SOST knockout attenuate downregulation of VEGF. All data were presents as mean ± SD, n = 6, ∗ *P* < 0.05; ∗∗ *P* < 0.01; ∗∗∗ *P* < 0.001;

We used the CRISPR/Cas9 system to construct SOST-Knockout (SOST ^−/−^) rats. The micro-CT scanning and H&E staining showed knockout of SOST yielded high bone mass phenotype compared with wild type (Fig. 7a, b). The micro-CT scan of femoral heads in SOST ^−/−^ showed more dense structure and gross appearance of femoral heads in SOST ^−/−^ exhibited fewer bleeding foci compared with wild type (Fig. 7d). In total, 7 of 12 rats in wild type group had obvious and visible signs of osteonecrosis according to histological analysis. However, none of the rats in the SOST ^−/−^ group showed signs of ONFH (Fig. 7e–g). The immunohistochemical staining of ALP and VEGF showed the decreased staining in wild type group and inhibitory effect of steroid could be neutralized by knockout of SOST (Fig. 7h–k).

### Bone metabolism was improved by knockout of SOST in rat model

**Fig. 8:**
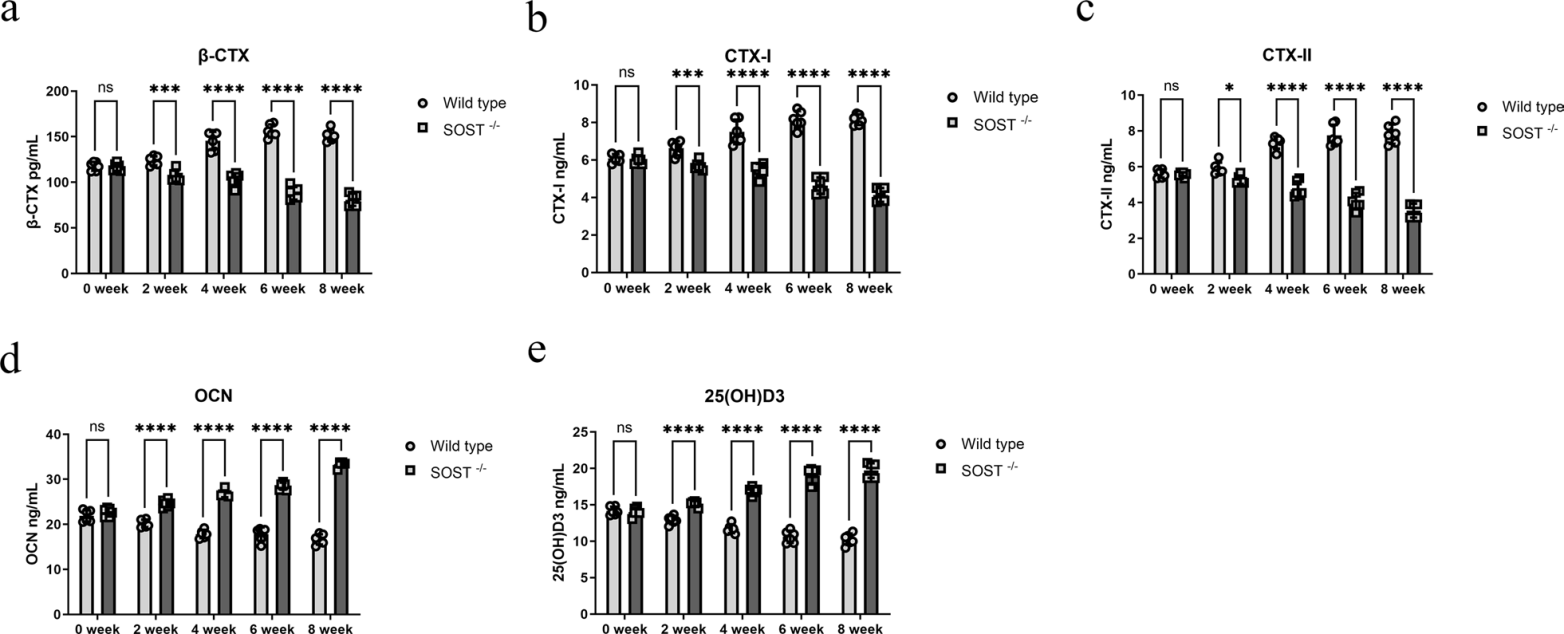
SOST knockout antagonized increased catabolism and decreased anabolism following steroid administration. **a**, **b**, **c**, **d** The serum catabolic level, β-CTX, CTX-I, CTX-II, was tested by enzyme-linked immunosorbent assays at different time points. **e **The serum anabolic level, OCN, 25(OH)D3, was tested by enzyme-linked immunosorbent assays at different time points. All data were presents as mean ± SD, n = 6, ns, not statistical significance; ∗ *P* < 0.05; ∗∗∗ *P* < 0.001; ****P < 0.0001

For investigating effect of sclerostin on bone metabolism after steroid administration, we collected blood every 2 weeks before sacrifice. Catabolic bone markers β-CTX, CTX-I, CTX-II showed no significant difference before steroid administration. Steroid administration could upregulate serum level of catabolic marker in wild type group and knockout of SOST antagonized this effect following 2, 4, 6, 8 weeks of steroid administration (Fig. 8a–c). Anabolic bone markers, OCN and 25(OH)D3 were downregulated in wild type group but not in SOST ^−/−^ group following 2, 4, 6, 8 weeks of steroid administration (Fig. 8d, e).

## Discussion

Clinically, about half of GC users develop various degree of ONFH but the mechanisms of its pathogenesis are yet to be fully elucidated (Koo et al. 2002). Although dexamethasone has been demonstrated its negative effect on osteoblasts, osteocytes and endothelial cells in vitro, the specific mechanism of dexamethasone in osteonecrosis remains unclear, especially the interaction of various cells in bone niche in the presence of dexamethasone. In our study, we found sclerostin accumulation in the necrotic area of the femoral head and identified the potential pathophysiological mechanism that dexamethasone stimulates osteocytes to secret sclerostin, which in turn produces a synergistic inhibitory effect with dexamethasone for osteogenesis and angiogenesis.

In the bone tissue, osteocytes accounts for 95% of the bone cells, act as the major bone sensor for transducing external mechanical signals and producing cytokines to maintain bone homeostasis (Bonewald LJJob, Bone mrtojotASf, Research M 2011; Zhou et al. 2015). When osteocytes undergo cell death, their secretions and metabolites also change. Enhanced osteocyte death is a key feature in many, but not all forms of bone diseases and is frequently observed in osteonecrosis, fracture, and inflammatory bone disease (Andreev et al. 2020). In osteonecrosis, previous study has reported the enhanced osteocyte death and the increased osteoclasts formation in necrotic area, but the blood vessel number decreased and the osteogenic activity impairment. In this study, we observed the impairment of ALP and VEGF staining in the necrotic areas, but also found significant deposition of sclerostin in the necrotic area. In vitro, we confirmed dexamethasone stimulation causes osteocytes to secrete more sclerostin, which may be the source of sclerostin in necrotic area. Additionally, in the process of osteocyte maturation, the sclerostin secreted by osteocytes will be deposited in the bone matrix (Marzin and Cormier-Daire 2020; Suen and Qin. 2016). When osteocytes death occurs, the absorption of bone matrix by osteoclasts will release a large amount of sclerostin, which may also be the source of sclerostin in the necrotic area.

Sclerostin is a 22.5 kDa secreted cysteine knot glycoprotein mainly produced by the mature osteocytes in bone tissue. In physiological state, the expression of sclerostin is regulated by a wide variety of factors, including local cytokines, hormones such as PTH and estrogen, and mechanical loading (Genetos et al. 2011; Keller 2005; Fujita et al. 2014; Winkler, et al. 2003). As a negative regulator of bone homeostasis, the increased level of sclerostin was observed in skeletal disease and bone loss. In GA-ONFH, according to our data from co-culture system, we could conclude that dexamethasone promotes sclerostin production by osteocytes and sclerostin is involved in the inhibitory effect of dexamethasone on the proliferation and differentiation of osteoblasts and vascular endothelial cells. Sclerostin is a potent inhibitor of Wnt signaling pathway by binding to its coreceptors, low-density lipoprotein receptor-related proteins 5 and 6 (LRP5 and LRP6). In the presence of sclerostin, Wnt-receptor interaction is inhibited, and β-catenin is phosphorylated by GSK-3β and targeted for ubiquitination and degradation via the proteosome pathway (Huang, et al. 2017). Dexamethasone administration could inhibit β-catenin signaling by activating GSK-3β with decreasing phosphorylation level, in line with previous study (Huang et al. 2017b). Co-culture with osteocytes further decreased the phosphorylation level of GSK-3β, followed by β-catenin degradation. As the cascade target of β-catenin, Runx2 and VEGF are major regulators of osteogenesis and angiogenesis (Yu et al. 2016; Wang, et al. 2014; Kong, et al. 2020). Wang et al. and Ma et al. respectively reported the downregulation of Runx2 and VEGF during osteonecrosis (Yu et al. 2016; Ma et al. 2010). We verified this phenomenon in our experiments both in vitro and in vivo, and the downregulated Runx2 and VEGF could be antagonized by reducing sclerostin expression in vitro and in vivo through Si-RNA or CRISPR/Cas9 technique.

Osteogenesis and angiogenesis are intimately connected and their tight coupling is very important to the physiological homeostasis of bone. In osteoporosis, the rapid transformation of bone metabolism is accompanied by decreased angiogenesis. During bone repair and regeneration, endothelial cells could enhance osteogenic differentiation of mesenchymal stem cells and osteoblasts via direct cell–cell contact. In contrast, growth of the vascular network is also regulated by signals provided by bone cells (Zhang et al. 2016). In our GA-ONFH model, wild type rats showed higher rates of necrosis and accelerated bone turnover. SOST knockout not only ameliorated the incidence of osteonecrosis, but showed a net gain in bone metabolism, that is bone formation exceeds bone resorption during bone development, growth and remodeling (Hu 2016; Migliaccio et al. 2007). Considering the inhibitory role of sclerostin on osteogenesis and angiogenesis, we speculate that sclerostin has an effect on both of the pathogenesis and repair process of GA-ONFH, but this conclusion still needs to be further verified.

Several limitations should be addressed in our study. Our results revealed that sclerostin secreted by osteocytes play an important role in GA-ONFH, but we could not exclude that other osteocyte secretions have a similar effect on GA-ONFH. Also, we were unable to verify in depth the effects of SOST due to limited resources.

It is worth noting that a commercial sclerostin antibody (romosozumab) had been approved for the treatment of osteoporosis in postmenopausal women with a high risk of fracture in 2019. Our study provides strong evidence that sclerostin played an important role in the development of GA-ONFH and blocking sclerostin secretion could improve bone turnover and ameliorate occurrence of osteonecrosis. Thus, we cautiously assume that usage of sclerostin antibody for patients at high risk of osteonecrosis or patients with osteonecrosis appears to be a viable treatment.

## Conclusion

In summary, our results demonstrated that sclerostin suppressed osteogenesis and angiogenesis through inactivating Wnt signaling pathway in GA-ONFH, which provided a potential treatment strategy for GA-ONFH.

## Supplementary Information

Additional file 1.

Additional file 2.

## Data Availability

The datasets used and/or analyzed during the current study available from the corresponding author on reasonable request.

## Abbreviations

GA-ONFH: Glucocorticoid-associated osteonecrosis of the femoral head
SOST: Sclerostin
GC: Glucocorticoids
SLE: Systemic lupus erythematosus
siRNA: Small interfering RNA
LPS: Lipopolysaccharide
MPS: Methylprednisolone
H&E: Hematoxylin and eosin
