# Supplementary figures and images for: SOST/Sclerostin impairs the osteogenesis and angiogesis in glucocorticoid-associated osteonecrosis of femoral head
Source: Mol Med. 2024 Sep 28;30:167. doi: 10.1186/s10020-024-00933-5 (PMC11439244; doi:10.1186/s10020-024-00933-5)

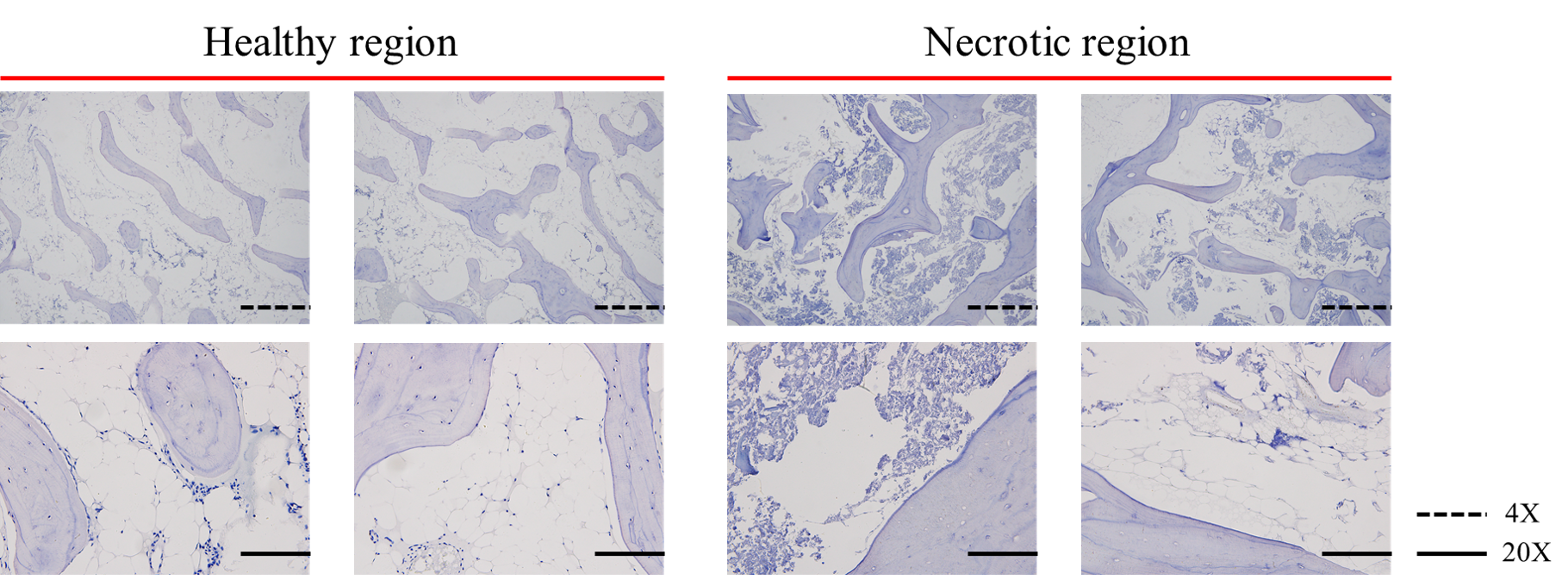

Supplement: Supplementary file 1 — Additional file 1. [file 10020_2024_933_MOESM1_ESM.tif]

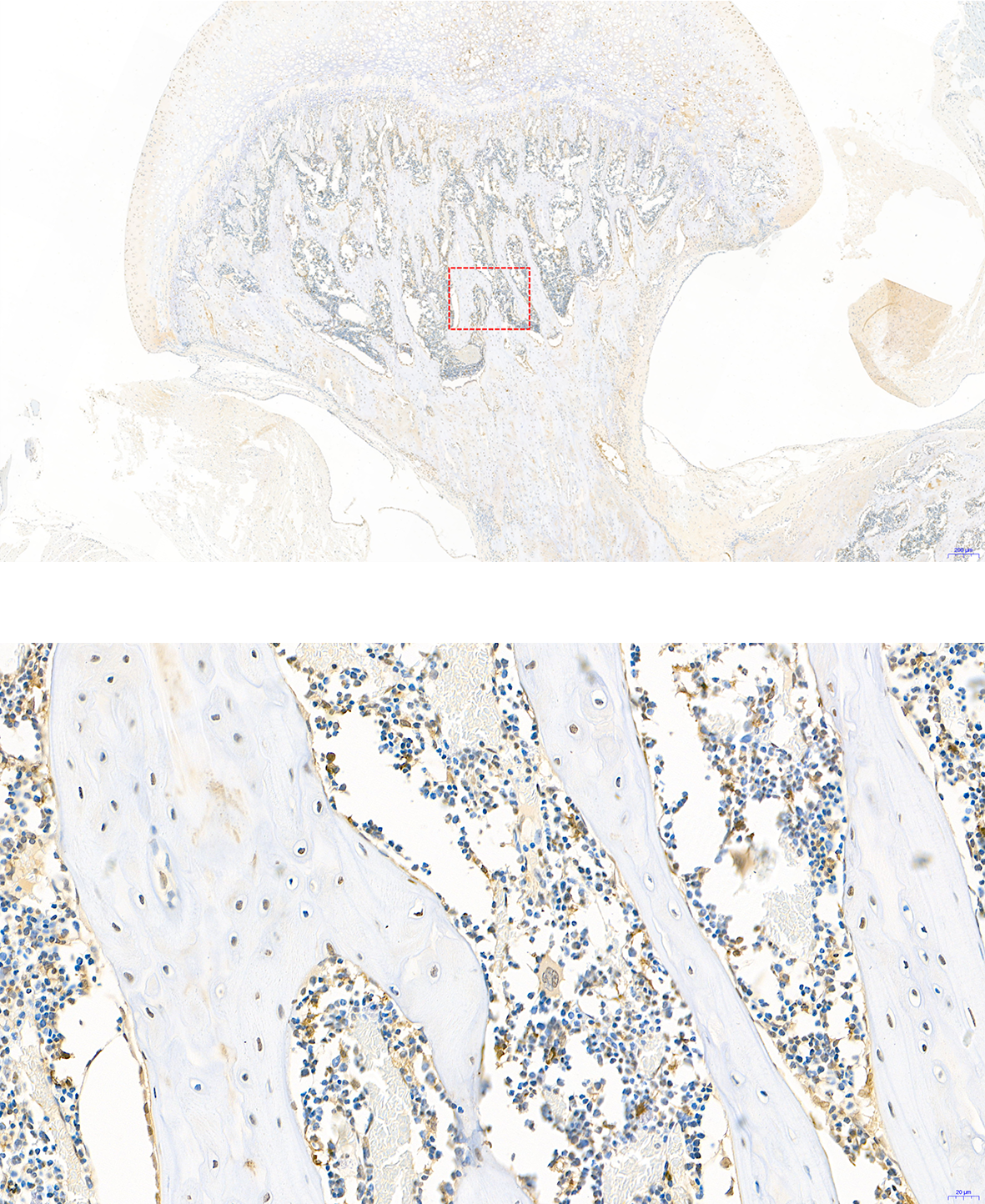

Supplement: Supplementary file 2 — Additional file 2. [file 10020_2024_933_MOESM2_ESM.tif]
